# Targeting Primary Motor Cortex (M1) Functional Components in M1 Gliomas Enhances Safe Resection and Reveals M1 Plasticity Potentials

**DOI:** 10.3390/cancers13153808

**Published:** 2021-07-28

**Authors:** Marco Rossi, Luca Viganò, Gugliemo Puglisi, Marco Conti Nibali, Antonella Leonetti, Lorenzo Gay, Tommaso Sciortino, Luca Fornia, Vincenzo Callipo, Marta Lamperti, Marco Riva, Gabriella Cerri, Lorenzo Bello

**Affiliations:** 1 Neurosurgical Oncology Unit, Department of Oncology and Hemato-Oncology, Università degli Studi di Milano, 20122 Milano, Italy; rossi.marco@gmail.com (M.R.); luca.vigano2@gmail.com (L.V.); guglielmo.puglisi@gmail.com (G.P.); marco.continibali@gmail.com (M.C.N.); antonellaleonetti80@gmail.com (A.L.); lorenzoggay@gmail.com (L.G.); tsciortino11@gmail.com (T.S.); vincenzo.callipo@yahoo.com (V.C.); lammar90@hotmail.it (M.L.); marco.riva@unimi.it (M.R.); 2 Department of Medical Biotechnology and Translational Medicine, Università degli Studi di Milano, 20147 Milano, Italy; luca.fornia@unimi.it (L.F.); gabriella.cerri@unimi.it (G.C.); 3 Laboratory of Motor Control, Department of Medical Biotechnology and Translational Medicine, Università degli Studi di Milano, 20147 Milano, Italy

**Keywords:** primary motor cortex, brain mapping, gliomas, intraoperative neurophysiology, functional organization

## Abstract

**Simple Summary:**

Primary-Motor-Cortex (M1) hosts two functional components, at its posterior and anterior borders, the first being faster and more excitable than the second. Our study reports a novel technique for the on-line identification of these functional components during M1 tumors resection. It reports for the first time the potential plastic reorganization of M1 and specifically how its functional organization is affected by a growing tumor and correlated to clinical, tumor-related factors and patient motor functional performance. It also shows for the first time that detecting the M1 functional architecture and targeting the two M1 functional components facilitates tumor resection, increasing the rate of complete tumor removal, while maintaining the patient’s functional motor capacity.

**Abstract:**

Primary-Motor-Cortex (M1) hosts two functional components, at its posterior and anterior borders, being the first faster and more excitable. We developed a mapping-technique for M1 components identification and determined their functional cortical-subcortical architecture in M1 gliomas and the impact of their identification on tumor resection and motor performance. A novel advanced mapping technique was used in 102 tumors within M1 or CorticoSpinal-Tract to identify M1-two components. High-Frequency-stimulation (2–5 pulses) with an on-line qualitative and quantitative analysis of motor responses was used; the two components’ cortical/subcortical spatial distribution correlated to clinical, tumor-related factor and patients’ motor outcome; a cohort treated with standard-mapping was used for comparison. The two functional components were always identified on-line; in tumors not affecting M1, its functional segregation was preserved. In M1 tumors, two architectures, both preserving the two components, were disclosed: in 50%, a normal cortical/subcortical architecture emerged, while 50% revealed a distorted architecture with loss of anatomical reference and somatotopy, not associated with tumor histo-molecular features or volume, but with a previous treatment. Motor performance was maintained, suggesting functional compensation. By preserving the highest and resecting the lowest excitability component, the complete-resection increased with low morbidity. The real-time identification of two M1 functional components and the preservation of the highest excitability one increases safe resection, revealing M1 plasticity potentials.

## 1. Introduction

Recent evidence in monkeys report a non-homogeneous functional organization of the primary-motor-cortex (M1), with the traditional somatotopic body divisions map [1,2,3,4] hosting two different functional components [5,6]: the anterior, with lower excitability and slower motor responses, and the posterior, with higher excitability and faster motor responses [6]. In monkeys, anterior-M1 neurons connect the spinal motoneurons indirectly, while posterior-M1 neurons connect monosynaptically [6,7]. Previous reports showed a similar rostro-caudal functional segregation in the human M1 hand-knob region [8,9], while no data on other body divisions, nor evidence on the effect of M1 lesions on this anatomo-functional compartmentalization were reported. In this respect, gliomas originating within M1 represent a challenge for neurosurgeons. To resect M1 tumors, surgeons must identify and preserve, using a brain mapping technique, the M1 neural structures essential for movement [10,11,12]. Standard mapping uses Low-Frequency (LF) or High-Frequency (HF) to identify essential sites within M1 [11,13,14,15,16,17,18], affording different degrees of tumor resection and permanent motor morbidity according to the clinical context [10,11,12,13,14,15,16,17,18,19,20,21]. LF affords a 50% rate of complete resection and 10% incidence of moderate to severe motor deficits; HF a 72.5% of complete resection and a 3.9% incidence of moderate motor deficits. HF-To5 is particularly efficient in tumors reaching the M1 surface and with well-defined borders, while in those with irregular borders and/or deep-seated it induces motor responses all over the precentral gyrus, preventing the identification of a safe entry zone and/or evoked motor responses since the earlier stages of the resection, affording only the achievement of a partial resection or a biopsy [10,11]. The achievement of a larger Extent of Resection (EOR), correlated to a better oncological outcome, stands on the ability to use the intraoperative neurophysiological tools to identify the essential sites within M1 at the cortical and subcortical levels [11,13,14,15,16,17,18]. A deep knowledge of the anatomo-functional organization of M1 is thus a mandatory background. Unfortunately, standard mapping fails in distinguishing between the anterior and posterior components [9]. Their different connectivity suggests a different role in motor control and a different effect of their lesions on motor performance [9], a crucial aspect for resection. Should the surgical lesion of some sectors result in transient rather than permanent deficits, the resection, based on this knowledge, could achieve higher efficacy. The lack of an adequate intraoperative technique to track the two components is therefore a surgical weak point, worsened by the fact that tumors, by their nature, induce the functional re-organization of M1. Whether clinical or tumor features affect the rostro-caudal functional segregation and how this reverberates on patient motor performance remains unsolved; how a neurophysiological intraoperative technique discriminating the two components would influence the chance of resection or patient morbidity is also unknown. As closely related aspect, a neurophysiological intraoperative technique discriminating the two components, beyond surgical purposes, would also represent a unique tool to directly investigate whether a tumor induces M1 functional neuroplasticity.

We here demonstrate that the intraoperative identification of the two M1 components requires the on-line measurement of quantitative neurophysiological parameters in addition to the qualitative approach (type of muscle activated) characterizing the standard technique [10,11,12,15,16,17], and, specifically: the minimum number of pulses needed for effective stimulation, Motor-Threshold, MEPs amplitude and latency. Our approach constitutes a novel “advanced motor mapping technique” allowing the identification of the two M1 functional components during surgical procedures [5]. Upon a description of the technical details, we report: how the rostro-caudal functional segregation at the cortical and subcortical levels is affected by patient clinical features, tumor imaging and histo-molecular parameters; the effect of the resection of one of the two components on motor performance; oncological and functional results obtained on M1 tumors resection. We show that the real-time identification of two M1 functional components and the preservation of the one characterized by the highest excitability one increases the rate of complete resection and maintains motor capacity, revealing M1 plasticity potentials.

## 2. Materials and Methods

### 2.1. Patients

A total of 102 glioma patients undergoing resection by the “advanced motor mapping technique” between 2017–2020 were included: 66 with tumors within M1, 28 with tumors involving CorticoSpinal-Tract (CST) but not M1, 9 with tumors outside CST-M1. Fifty-one patients with tumors within M1, operated with the standard mapping technique (January–December 2016), were used to compare the Extent-Of-Resection (EOR). Clinical, imaging and histo-molecular features of patients and tumors were collected. Patients gave a formal consent to procedures and to the study (IRB-1299). 

### 2.2. Surgery and Anesthesia

During procedures, the craniotomy exposed the tumor area and, when needed, a large part of M1. Cortical motor mapping was first performed to disclose the M1 functional architecture, to identify the safe entry zone for the corticectomy. Subcortical motor mapping guided the resection by identifying the two M1 fiber components and the margin of resection [10,15,21]. All surgeries were performed under general anesthesia, using propofol and remifentanil, and titrating the level of drugs to EEG and ECoG signals, in order to avoid any burst suppression and maintaining a continuous level of brain activity. 

### 2.3. Monitoring Tools

ElectroEncephalography-EEG and ElectroCorticography-ECoG signals provided a real-time monitor of the depth of anesthesia and the occurrence of seizures/afterdischarges. EEG was recorded since the beginning of anesthesia and in parallel to ECoG, throughout the surgery. A multichannel recording EMG system monitored 24 contralateral and ipsilateral muscles (Text S1) [10,15], allowing us to inspect: free-running-background-EMG activity; Motor Evoked Potentials-MEPs to brain mapping stimulation; MEPs evoked by M1 Direct-Cortical-Stimulation (To5-monitoring technique to assess M1-CST integrity) elicited by transcranial electrodes from incision to closure, or strip electrodes during the resection time (small 4-contact-strips placed in the surgical field [10,15]).

### 2.4. Advanced-Motor Mapping

Motor mapping was performed with High-Frequency stimulation (monophasic stimulus, 500 ms pulse duration, ISI = 3–2 ms) delivered by a monopolar probe (straight tip, 1.5 mm diameter) combining the classical 5-shocks-mode (HF-To5) and the novel bistim-mode (HF-To2). 

#### 2.4.1. Cortical Mapping

**Figure 1:**
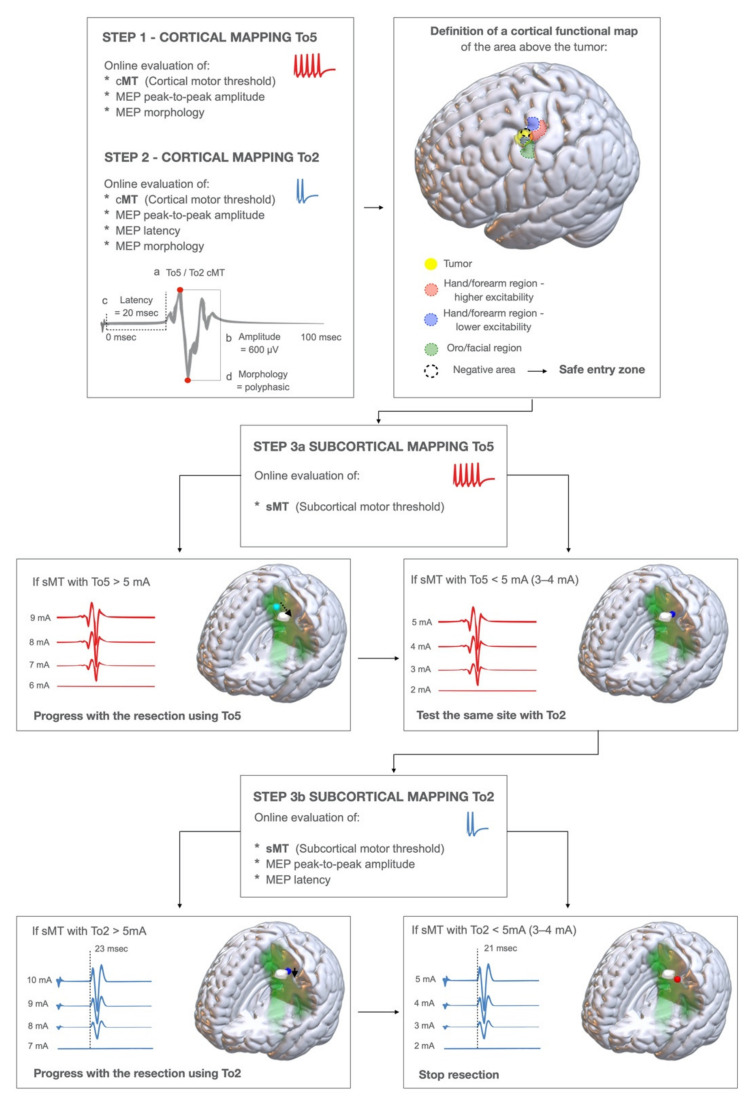
Steps of the brain mapping technique to detect the two functional components of M1. STEP1: a cortical mapping with HF-To5 paradigm is performed to explore the cortical area of interest. At each site (a) the cortical motor threshold (cMT), (b) the MEP peak-to peak amplitude, and (d) the MEP morphology are evaluated. See Figure S1 for practical insights on how to evaluate the peak-to-peak amplitude and MEP morphology. The Cortical Motor Threshold is defined as the lowest current intensity which is evoking the lowest MEP response. STEP2: the same sites are explored again with HF-To2, evaluating at each site: (a) the cMT, (b) the MEP peak-to-peak amplitude, (c) the MEP latency (evaluated from the first pulse artifact), (d) the MEP morphology. By performing this, we defined the cortical functional map, identifying: 1. The area of higher excitability, 2. The area of lower excitability, 3. The safe entry zone: the area where no MEPs are evoked (at current intensity > 20 mA, at HF-To2) or the area of lower excitability (see text). STEP3a: subcortical motor mapping initially performed with HF-To5. A continuous motor mapping is performed by placing the monopolar probe in the close vicinity of the resection site (suction) and coupled with it. At each site, the subcortical-MT (sMT) is continuously evaluated. If the sMT is > 5 mA, the resection is continued until sMT < 5 mA; in this case, the same site is explored by HF-To2 STEP3b: subcortical motor mapping with HF-To2; at each resection site, we evaluated (a) the sMT, (b) the MEP peak-to-peak amplitude, (c) MEP latency, (d) the MEP morphology. If the sMT > 5 mA, the resection is continued until the sMT is < 5 mA, and when sMT is 2–3 mA the resection is stopped.

**Figure 2:**
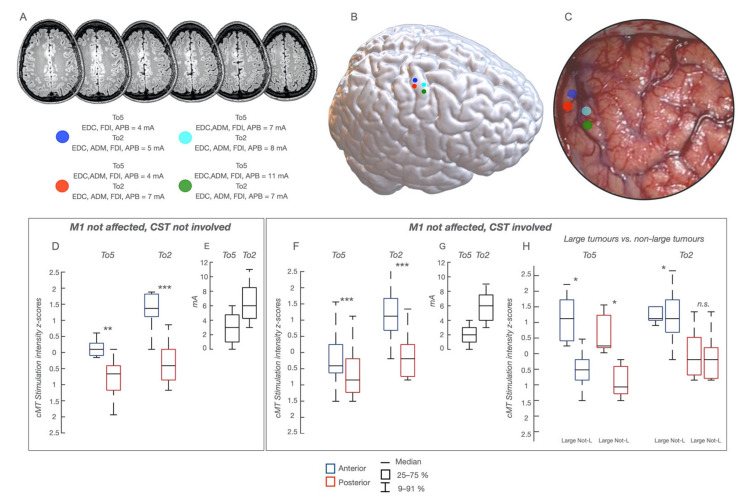
Functional Map in patients with M1 intact and CST not involved or involved at variable extent, whose cortical functional map is presented in a representative patient with a glioma involving the non-dominant hemisphere; the lesion is visible in (**A**), sequential axial FLAIR images and a poorly defined mass (low-grade) involving the pre-SMA region. The sites of HF-To5 and HF-To2 stimulation in the hand-knob region are visible as colored dots superimposed to the NMI space (**B**) and to the intraoperative field picture (**C**). For each dot, the cMT (quantitative parameter) at HF-To5 and HF-To2 and the pattern of muscle activated (qualitative parameters) are reported. (**D**) Comparison of cMT obtained with HF-To5 and HF-To2 for the stimulation of the anterior and posterior region of the hand-knob area in the 9 patients with M1 and CST not involved; cMT are expressed as 25–75% median (rectangle) and 9–91% (edge of the box), along with the delta (**E**); * *p* < 0.5; ** *p* < 0.01; *** *p* < 0.001. (**F**) Comparison of cMT obtained with HF-To5 and HF-To2 stimulation of the anterior and posterior region of the hand-knob area, in the 28 patients with M1 intact and the CST involved; cMT are expressed as 25–75% median (rectangle) and 9–91% (edge of the box), along with the delta (**G**); * *p* < 0.5; ** *p* < 0.01; *** *p* < 0.001. (**H**) Comparison of cMT at the anterior and posterior borders of the hand-knob area in the 5 patients of the previous group harboring a large tumor and in the remaining 23 patients. The cMT are higher in the first sub-group of patients (large tumors) with HF-To5 but not with HF-To2.

HF-To5 and HF-To2 were combined to disclose, in the exposed cortex, the anterior or posterior M1 border regions to establish the safe entry zone for the corticectomy. After dura opening, the cortex in the tumor area was first explored with HF-To5: at each stimulated site; the current intensity was initially set at 15 mA, and when a stable motor response was obtained, the intensity was decreased to the cortical-Motor-Threshold (cMT). The cMT was defined as the lowest current intensity inducing the lowest detectable MEP (at a 50 µm amplification scale). The type of MEPs (pattern of muscles activated) evoked at the cortical-Motor-Threshold (HF-To5 cMT) was identified and recorded. To depict the M1 functional subdivision, at each site, the cMT was initially identified with HF-To5; the same site was re-explored with HF-To2 to find the corresponding cMT. The current intensity was set at 15 mA, and when a stable MEP was elicited, it progressively decreased to cMT (HF-To2 cMT). The pattern of muscles activated at HF-To2 cMT (qualitative parameter), the MEP amplitude and the latency (Figure 1, Step1, Step2; Figure 2) obtained with HF-To2 (quantitative parameters) were compared on-line, to identify sites at higher or lower excitability.

InterStimulus-Interval = 3 ms was set for exploring the hand-knob and the mesial portion of M1 (leg-area), ISI = 2 ms for the dorsal portion of M1 (face-area), with the reference electrode placed close to M1, at the mesial border of the flap. We also mapped the used anodal stimulation, which occasionally switched to cathodal (in case of unstable responses). 

#### 2.4.2. Subcortical Mapping

In each area of interest, subcortical mapping aimed at identifying fibers with different functional properties coherent with the cortical components, to guide the identification of resection borders. Both HF-To5 and HF-To2 were continuously applied (catodal set-up) to identify the different fibers originating from M1 components. To ensure a continuous subcortical mapping, the monopolar probe was kept by the assisting surgeon in the surgical field, very close to the site of resection, in order to detect any motor response. Upon identification of a motor response, a current-intensity-curve was always performed to find the sMT, allowing for a reliable estimation of the distance from M1 corticofugal fibers. The same parameters estimated at the cortical level were used subcortically to infer to which of the two cortical components the fibers stimulated could be ascribed: the muscle pattern of MEPs, Motor-Threshold intensity, MEPs amplitude and latency. The HF-To2 MEPs amplitude and latency were calculated on-line (Figure 1, Step 3a–3b). Technically, mapping was initially performed with HF-To5, with the current intensity set at 10 mA. When a stable MEP was found, the current intensity was progressively decreased to find MEPs at threshold-sMT, at each site: when a MEP was evoked with sMT < 4 mA, stimulation was shifted to HF-To2, to find the corresponding sMT (expected to be higher than HF-To5 sMT, given the lower number of pulses). When HF-To2 evoked a MEP with intensity < 5 mA, then MEP latency, amplitude and morphology were assessed, and the intensity decreased until sMT (3–4 mA), set as the intensity estimating the minimum safe distance to the essential fibers defining the limit of resection.

Two M1 cases are shown in Video S1 (a case with a “normal” cortical and subcortical map) and Video S2 (a case with a “distorted” subcortical map), reporting the practical hints of the technique and the high degree of interaction between surgeon and neurophysiology-technician, an essential requisite for its correct application.

### 2.5. Imaging

The pre-operative MRI protocol (Siemens-Magnetom-Verio-3.0) included: axial-three-dimensional-(3D)-FLAIR; post-Gd-three-dimensional-T1-weighted; DWI-ADC diffusion-weighted imaging. Patients underwent post-operative-MR (volumetric-FLAIR and post-GdT1-weighted) both within 48 h and 2 months to estimate EOR [10,15,21]. An immediate post-operative-DWI was performed to evaluate ischemia [10,12].

### 2.6. Neurophysiological Data Analysis

**Figure 3:**
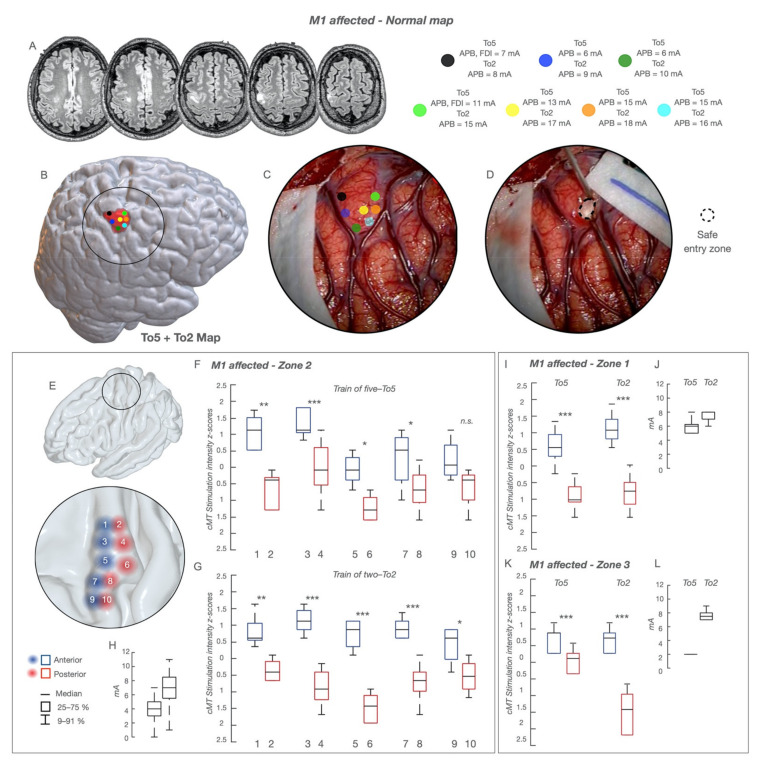
“Normal” functional cortical map in the group of 66 patients with tumors within M1. The functional cortical map is presented in a representative case of glioma involving the non-dominant hand-knob; the lesion is visible in (**A**), sequential axial FLAIR images, as a poorly defined mass (low-grade glioma) involving the hand-knob region of M1. The cortical area exposed (circle line), the sites stimulated with HF-To5 and HF-To2 (colored dots) and the tumor area (pink volume) are reconstructed in an NMI space and shown in (**B**). The picture of the intraoperative field along with the location of the stimulated sites (colored dots) superimposed to the surgical field is shown in (**C**). The entry point where the resection was started is shown in (**D**) as a dotted circle. For each dot, the cMT (quantitative parameter) at HF-To5 and HF-To2 and the pattern of muscle activated (qualitative parameters) are reported; when the same sites were stimulated with HF-To2, only APB MEPs were evoked at the anterior bank (cMT 15–17 mA) and at the posterior one (cMT 8–10 mA). A clear functional segregation was documented between the anterior (lower excitability) and posterior (higher excitability) regions. The safe entry zone (**D**) corresponded to the area of lower excitability. (**E**) The hand-knob region was divided based on 10 stimulated sites (1,3,5,7,9 covering the anterior region—red circles; 2,4,6,8,10 covering the posterior region—blue circles); in each site the cMT (absolute value, mA) at HF-To5 (**F**) and HF-To2 (**G**) was normalized within the subject (*Z*-score) to allow for a comparison between the anterior and posterior regions with each paradigm. The same procedure was applied for the other body division, lower limb (zone-1) (**I**) and face (zone-3) (**K**). cMT are expressed as 25–75% median (rectangle) and 9–91% (edge of the box), along with the delta (**H**,**J**,**L**); * *p* < 0.5; ** *p* < 0.01; *** *p* < 0.001.

For each cortical and subcortical site stimulated, on-line qualitative (muscles activated by HF-To5 or HF-To2) and quantitative parameters (cMT/sMT, MEPs amplitude, MEPs latency) were evaluated: MEP amplitude from peak-to-peak; latency calculated (on MEPs obtained with HF-To2) from the onset of stimulus artifact and MEP. For subcortical sites, MEPs morphology assessed the occurrence of polyphasic vs. monophasic responses, the latter representing a warning sign. All recorded neurophysiological data were stored for an off-line analysis. During surgery, mapping was videorecorded and the MRI coordinates of all stimulated sites were acquired by neuronavigation. For each patient, all site positions were registered to the brain volume (cortical surface and subcortical space) by Freesurfer manufacturer, city, state abbrev. if Canada of USA, country; the results were downloaded onto Brainstorm (a MATLAB toolbox; Tadel, Baillet, Mosher, Pantazis, & Leahy, 2011), obtaining a 3D-MRI reconstruction of each patient MR with all the sites marked as a scout. This was co-registered in the NMI-space [9,22,23]. In “normal”-map patients, the subdivision of the cortical hand-knob region was designed (Figure 3) based on 10 stimulated sites (1,3,5,7,9 covering the anterior region; 2,4,6,8,10 covering the posterior region); in each M1 site the cMT (absolute value/mA) at HF-To5 and HF-To2 was normalized within the subject (*Z*-score) to allow for a comparison between the anterior and posterior regions with each paradigm [9,22,23]. In “distorted”-map patients, in the absence of an anterior and posterior subdivision, the highest and lowest cMT in each patient obtained with HF-To5 vs. HF-To2 were selected. Values were normalized within the subject (*Z*-score) to assess the preservation, despite the subversion of the architecture of the two functional components [9,22,23,24].

### 2.7. Clinical Factors Analyzed

The clinical factors at admission included: age, gender, clinical history, presenting symptoms, previous treatments, type and number of seizures and seizure control, and current and previous pharmacological therapies. A neurological assessment (MRC evaluation) was performed at admission, 1 week after surgery, followed by 1- and 2-month follow-ups. An ARAT (Action-Research-Arm-Test) evaluation in patients with a tumor affecting zone 2 was also performed. The rehabilitation requirements were recorded. The tumor imaging variables, deductible from a conventional pre-operative-MR, included volume, zone location, side, borders, presence of contrast enhancement, outcrop of M1 cortex, extension outside M1 cortex [10,12,25]. The tumor volume was calculated using the Smartbrush software from Elements BrainLab (Munich, Germany); for contrast-enhancing lesions, the contrast-enhancing portion of the tumor (target of resection) was measured [10,12,25]. For zone location, M1 was divided into three zones (zone 1: lower limb; zone 2: upper limb; zone 3: face) [10,12]. Tumor borders were defined as well-defined or irregular [10,25], using post-contrast imaging for contrast lesions or FLAIR for non-enhancing. The outcrop of M1 was defined as present when the tumor was reaching that area of the cortex, either on coronal and/or sagittal FLAIR for non-enhancing lesions, or on T1-post-contrast images for enhancing lesions [10]. Regarding the extension of tumors within M1, only tumors with over 75% of their mass involving M1 were included; in cases of large tumor volumes, the tumor could extend anteriorly toward the dorsal-premotor or the supplementary-motor cortex or posteriorly toward the primary-sensory cortex. The extension was categorized accordingly [10,12]. The histological variables included histology and molecular profile (IDH-status, codeletion). The EOR calculation was performed on immediate post-operative MR (within 48 h), targeting post-contrast MR for enhancing lesions or FLAIR for non-enhancing lesions. EOR corresponded to the percentage of volume resected with respect to the preoperative volume: (preoperative volume–postoperative volume)/preoperative volume. Surgical resection was categorized as: (i) total resection (postoperative volume = 0 cm^3^), (ii) sub-total resection (postoperative volume 0 < 5 cm^3^) and (iii) partial resection (postoperative volume > 5 cm^3^) [10,12,19,21,26].

### 2.8. Statistics

The analyses were performed using the SPSS software, version 22.0 (IBM, Chicago, IL, USA). For categorical data, Pearson-chi-square (multiple categories) or Fisher-exact (2 categories) tests were reported. One-way ANOVAs were used for comparisons of continuous variables when comparing 2 or more groups. The predictive value of the significant variables from bivariate analysis was further assessed by multiple binomial logistic regression. Stimulation-intensity values were standardized within stimulation paradigms (HF-To5/HF-To2).

## 3. Results

### 3.1. Advanced Mapping Techniques Reveal a Remodeling of M1 Architecture

#### 3.1.1. Cortical Level. Data Were Clustered in Three Groups Based on the Degree of Tumor Involvement of M1 and/or CST

1. Tumor outside M1 and CST

In these patients (*n* = 9, Table S1) the technique depicted a well-defined cortical somato-topic organization with a clear segregation of the anterior and posterior regions in each body division. Within the hand-knob, HF-To5 cMT were higher (5–11 mA; median 9 mA) at the anterior compared to the posterior border sites (3–9 mA; median 5 mA). At the same posterior sites, cMT HF-To2 ranged between 5 and 13 mA (median 5 mA) and the average MEP-latency was 23 ms (APB, ADM, FDI), while at the anterior border MEPs required higher cMT (13–18 mA; median 16 mA) and were slower (average latency = 25–26 ms). cMT values over the hand-knob clustered along a rostro-caudal segregation, evident when comparing in each patient the cMT absolute values at HF-To5 vs. HF-To2 in the anterior and posterior regions and, at the population level, the cMT values expressed as *Z*-scores (Figure 2D,E). Conclusively, in tumors outside M1/CST, both paradigms depicted a rostro-caudal excitability difference, clearly segregated only with HF-To2.

2. Tumor affecting CST but not M1

In these patients (*n* = 28, Table S1), tumors were located anteriorly (*n* = 19) or posteriorly (*n* = 2) to M1, or in the insular lobe deeply affecting the CST (*n* = 7). Results were superimposable to the previous group (group-1): the anterior and posterior components were better depicted with HF-To2 (Figure 2A–C,F,G). Only in 5 cases with large tumors were cMT values higher, on average (Figure 2H).

**Figure 4:**
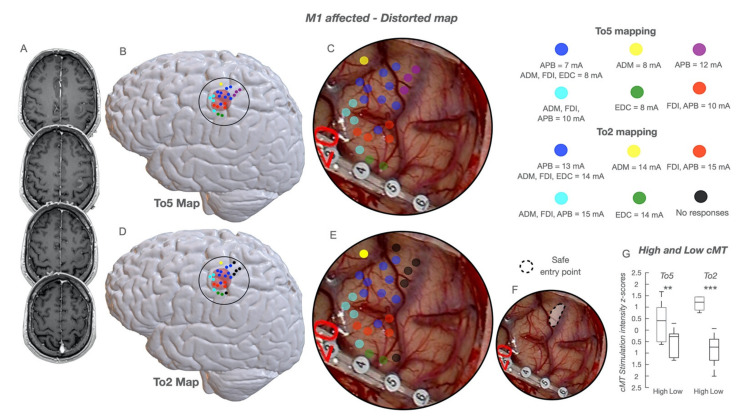
Distorted functional map in patients with M1 involved. The functional cortical map is presented in a case of glioma involving the hand-knob region in the dominant hemisphere; the lesion is visible in (**A**), sequential axial post GD T1 weighted images, as a well-defined patchy enhancing mass involving the hand-knob region of M1. The cortical area exposed (circle line), the sites stimulated with HF-To5 (colored dots) and the tumor area (pink volume) are reconstructed in an NMI space and shown in (**B**) (To5 Map). The cortical area exposed (circle line), the sites stimulated with HF-To2 (colored dots) and the tumor area are reconstructed in an NMI space and shown in (**D**) (To2 Map). The picture of the intraoperative field along with the location of the stimulated sites (colored dots) by using HF-To5 superimposed to the surgical field is shown in (**C**); a cortical strip is crossing the inferior part of the motor cortex (at the inferior border of the hand-knob region); HF-To5 evoked MEPs at variable cMT (ranging between 7 and 12 mA) without a functional segregation between the anterior and posterior regions. The picture of the intraoperative field along with the location of the stimulated sites (colored dots) by using HF-To2 superimposed to the surgical field is shown in (**E**); HF-To2 evoked MEPs at variable cMT (ranging between 13 and 15 mA) without a functional segregation between the anterior and posterior regions; however, a region in which no MEPs were evoked at a current intensity of 20 mA was detected in the superior and posterior regions of the motor cortex (no response, black dots); this region was used as a safe entry zone in which to perform the corticectomy and to start the resection (shown in (**F**) as a dotted circle line, safe entry point). In “distorted-map” patients, in the absence of an anterior and posterior subdivision, the highest and lowest cMT in each patient obtained with HF-To5 vs. HF-To2 were selected (**G**); the values were then normalized within the subject (*Z*-score) to assess the preservation, despite the subversion of the architecture, of the two different functional components. Cumulative cMT are expressed as 25–75% median (rectangle) and 9–91% (edge of the box); ** *p* < 0.01; *** *p* < 0.001.

3. Tumor originating within M1
  In these patients (*n* = 66, Table 1), two different architectural configurations emerged:
  (a) “*normal*”*cortical map*. HF-To5 and HF-To2 evoked MEPs over the entire M1, depicting a “well-defined” somatotopic organization, from the mesial (lower-arm MEPs) to the dorsal division (face-tongue MEPs) (Figure 3). Similarly to previous groups, the anterior and posterior components were disclosed by both HF-To5 and HF-To2. In the hand-knob, HF-To5 cMT were higher at the anterior (6–12 mA; median 11 mA) compared to the posterior border (3–10 mA; median 6 mA). In the same posterior sites, HF-To2 cMT ranged between 4 and 10 mA (median 6 mA), with an average MEP-latency of 23 ms in hand muscles (APB,ADM,FDI); when applied at the anterior border, HF-To2 required a higher current intensity (12–17mA; median 14 mA) to elicit slower hand MEPs (average latency = 25–26 ms) (Figure 3). HF-To2 at cMT evoked fewer muscles (1 or 2) compared to HF-To5 (3 to 4) at cMT applied at the same site (Figure S1). The rostro-caudal segregation emerged also in other body divisions (Figure 3E–H,I–K), where both paradigms required higher currents compared to that of the hand knob (average cMT for face-tongue MEPs + 2 mA; average cMT for proximal upper-arm or lower-limb MEPs + 3–5 mA). Here, again, HF-To2 offered a better depiction of the rostro-caudal difference (Figure 3H,J,L).
  (b) “*distorted*”*cortical map*. Both HF-To5 and HF-To2 evoked MEPs over all M1, but its functional organization was clearly distorted (Figure 4). HF-To5 failed in identifying excitability differences between the anterior and posterior borders, eliciting MEPs at the same cMT (4–18 mA; median 9 mA) over all the gyrus, suggesting the absence of the rostro-caudal segregation (Figure 4B,C). Only HF-To2 (4–18 mA; median 7mA) successfully disclosed sites where MEPs were evoked at different cMT: a rearranged cortex with a mixed distribution of sites at lower and higher cMT emerged (Figure 4D,C). In the absence of an antero-posterior subdivision, the analysis of the highest and lowest cMT in each patient obtained only with HF-To2 confirmed the preservation of the two different functional components in a subverted architecture (Figure 4G). As the complexity increased, the cortical somatotopic distribution was also intertwined, with hand muscle MEPs (flexors) evoked also in the dorsal or mesial portion of M1, intermingled with leg and face responses and with the same cMT. Similarly, in zone-1 tumors, lower-limb MEPs were evoked in the hand-knob region. Here, HF-To5 failed to detect the clear safe entry zone, identified only by HF-To2 (Figure 4F).

The average duration of cortical mapping was similar (11 ± 2 min in “normal” map; 13 ± 3 min in “distorted” map).

#### 3.1.2. Subcortical Level. for Clinical Reasons, Data Were Solely Acquired in the 66 Patients with Tumors within M1. Again, 2 Patterns Emerged

a *“Normal”subcortical map*. At the subcortical level, HF-To5 evoked MEPs with similar sMT proximal to both the anterior and posterior banks; HF-To2 evoked MEPs at the lowest sMT and shortest latency in subcortical sites near the posterior bank compared to sites near the anterior bank requiring higher intensities (Video S1). Precisely, at the posterior bank, HF-To5 evoked MEPs at sMT < 4 mA since early stages of the subcortical phase. Here, HF-To2 evoked MEPs at sMT > 5 mA, and the intensity was decreased until an sMT of 3 mA: MEPs latency ranged between 24 and 20 ms (depending on the depth of the site) for hand muscles, 32 and 28 ms for lower limb muscles and 12 and 20 ms for face muscles. At the anterior bank, HF-To5 evoked MEPs at sMT < 5 mA (very similar to the posterior bank), but HF-To2 sMT was >10 mA (range = 10–17 mA), amplifying the difference with the posterior bank. MEPs latency ranged between 26 and 24 ms for hand and 34–30 ms for lower limb muscles. Only HF-To2 identified an anterior and posterior component.
b *“Distorted”subcortical map*. HF-To5 elicited MEPs at sMT < 4 mA in most sites. Only HF-To2 identified sites where MEPs were evoked at the lowest sMT and shortest latency in different gyrus location, either pushed anteriorly or laterally to the tumor bulk, depending on each individual case and allowing us to continue and complete the resection (Video S2). In this case, sites where HF-To5 elicited MEPs at the lowest sMT (<4 mA) and HF-To2 generated MEPs at the lowest sMT (3–4 mA) were intermingled with sites where MEPs were evoked by HF-To5 again at sMT < 4 mA but by HF-To2 at higher current intensities (>10mA). HF-To2 revealed the preservation of two components in a distorted organization with loss of anatomical reference and somatotopy.

The average duration of mapping was 18 ± 2 min in *“normal”subcortical map* and 26 ± 4 min in *“distorted”subcortical map*.

### 3.2. M1 Functional Cortical/Subcortical Architectures and Clinical Variables

**Figure 5:**
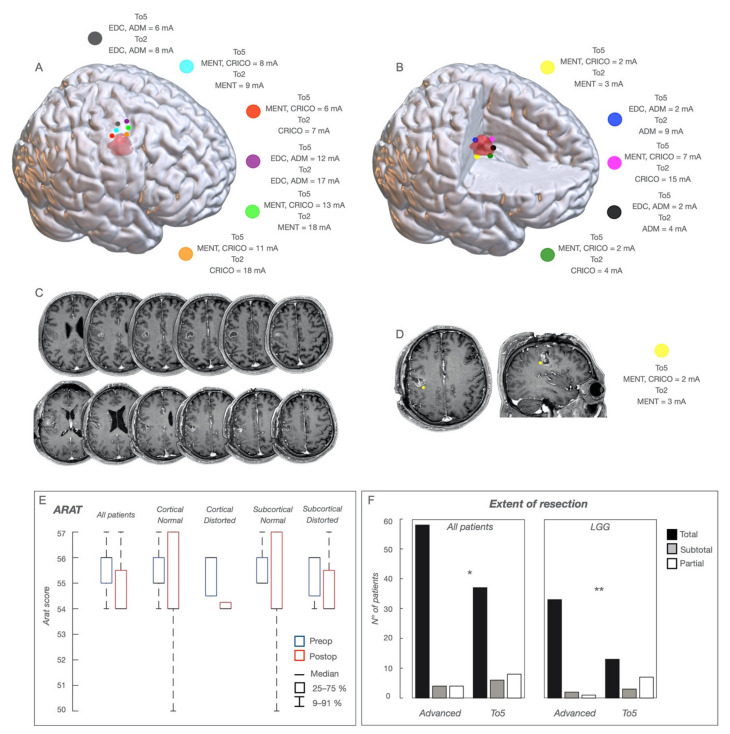
Functional Maps and Resection. A case of glioma involving, in the non-dominant hemisphere, the vPM and the face area of M1 (zone-3), in a representative patient who experienced several left face focal seizures. The location of the tumor is shown in (**C**) (sequential axial post-GDT1-weighted images); the tumor appeared as a solid, well-demarcated mass with contrast enhancement nodules. The patient was submitted to a sleep surgery with the aid of the advanced brain mapping technique. A cartoon depicting, in the NMI space, the cortical functional map is shown in (**A**). The tumor volume is in pink. The stimulated sites are shown as colored dots. At the cortical level, HF-To5 and HF-To2 evoked face (Mentalis and Cricothyroid) MEPs at variable cMT (To5 ranging between 6 and 13 mA; To2 ranging between 7 and 18 mA) with a clear functional segregation between the anterior and posterior regions. At the subcortical level (**B**), sites evoking hand or face muscle MEPs at the lowest sMT were identified. Those with the lowest sMT at HF-To5 and HF-To2 (yellow, blue, black and dark green) were used as the margin of resection; sites with the lowest sMT at HF-To5 but with sMT at HF-To2 > 4 mA (representative site indicated by a magenta dot) were removed. The post-operative MR is shown in (**D**) (sequential axial post-GD-T1 weighted images). A representative location of a functional site identified by subcortical mapping is shown at the posterior and inferior margin of the tumor as a colored yellow dot, superimposed to the preoperative axial and coronal post-GD-T1-weighted images. HF-To5 evoked MEPs of Mentalis and Cricothyroid at an sMT of 2 mA; HF-To2 evoked only cricothyroid MEPs at an sMT of 3 mA, indicating that these fibers belong to the posterior functional component. A histo-molecular diagnosis showed a grade-III IDH-wildtype glioblastoma, TERT Intact. (**E**) Action-Research-Arm-Test (ARAT) evaluation score performed in the group of patients with tumors involving the hand-knob region of M1; the pre- (preop) and post-operative (45 days of surgery) (postop) scores are presented for the whole group (all patients), patients with normal cortical map, patients with normal subcortical map, patients with distorted cortical map and patients with distorted subcortical map. Scores are presented as 25–75% median (rectangle) and 9–91% (edge of the box). (**F**) Comparison of Extent of resection achieved in the 66 patients with tumors involving M1, in whom resection was guided by the advanced motor mapping technique (Advanced) and in the 51 patients with similar tumors, in whom resection was guided by the traditional To5 motor mapping technique (To5); the same comparison is presented for patients with low-grade gliomas (LGG); data are expressed as EOR classes: Total resection, subtotal, or partial; * *p* < 0.5; ** *p* < 0.01.

The analysis was run in patients with tumors within M1 (Table 1). A “normal”cortical map was found in 36 cases (54.5%), and the “distorted”cortical map in 30 (45.5%) (Table 1); (a) a “normal”subcortical map was found in 32 cases (48.5%), and a “distorted” subcortical map in 34 (51.5%). The cortical and subcortical “normal” maps and the cortical and subcortical “distorted” map always matched, except for 2 patients (“distorted”subcortical map associated with “normal”cortical map). “Distorted”cortical maps were associated with previous treatment, seizure control and subcortical pattern. “Distorted”subcortical maps were associated with previous treatment, seizure control and AED uptake (Table 2 and Table 3). Cortical/subcortical maps were independent of tumor volume (Table 3). Most patients showed a normal motor capacity at presentation, and zone-2 patients had normal ARAT scores (Figure 5). Pre-operative motor performance was not associated with cortical/subcortical maps or histo-molecular features (Table 3).

### 3.3. Disclosing M1 Functional Architecture Increases Resection

In the 66 patients with tumors within M1, the corticectomy was performed in the area of lowest cortical excitability identified by HF-To2 (Figure 3C,D and Figure 4E,F). An unresponsive area (no MEPs at intensity > 20 mA) was identified in 21 cases only (31.8%), while in the others it was related to the cortical map: in “normal” maps, the lowest cortical excitability was found at the anterior border; in “distorted” maps, the location depended on each individual case. During the subcortical resection, the identification of sites with different functional properties was mandatory to guide the resection by establishing the margins. Technically, a site in which MEPs were evoked by HF-To5 at the lowest current intensity (<4 ms) was re-explored by HF-To2 to find sMT. The resection was continued until an sMT of 3–4 mA: MEPs amplitude and latency were assessed. Sites where MEPs were evoked by HF-To2 at sMT intensity > 4 mA were removed (including those where HF-To5 evoked MEPs at sMT < 4 mA). The resection stopped when HF-To2 evoked MEPs at sMT = 3 mA, and the latency values were those previously reported.

The mapping strategy was particularly useful in lower grades (Figure 5F); independent of the normal or distorted arrangement, a safe entry zone was identified, pursuing resection until the subcortical sites where MEPs were evoked with the lowest current and the shortest latency, affording a high percentage of complete resection. In high-grade tumors, the technique identified the safe entry zone in case of “distorted”cortical maps (37.5% of cases) or increased zone-3 tumors resection (Figure 5A–D).

A total resection was achieved in 58 patients (88%) (Table 1 and Table 2) and associated solely with tumor volume (Table 2 and Table 3), independent of the cortical or subcortical map encountered. Compared to the standard To5-technique, the mapping strategy increased the complete resection (Table S2), particularly in lower grades (Figure 5F).

### 3.4. Resection Strategy and Functional Outcome

The resection strategy preserved the cortical and subcortical sites with the highest excitability. Motor performance declined in the acute post-operative period in 51 (77.3%) patients: MRC scored 3 in 21 (31.8%) patients, 4 or 5 in the remaining 68.2% (Table 2). Patients with zone-2 tumors, despite the resection of the lowest excitability component, maintained individual digit movement and/or elbow flexion; rather, it was the performance of motor tasks that required a long and close visual guidance (Videos S3 and S4). Acute deficits were associated with histo-molecular features and borders (irregular), but were independent of “normal” or “distorted” cortical or subcortical maps. MRC = 5 was associated with a long clinical history and well-defined borders (Table 2 and Table 3).

Most patients recovered in 1 month (Table 1); 22 patients had rehabilitation (33.3%), lasting less than 3 weeks on average. Rehabilitation was associated with shortest clinical history (<2 months), pre-operative deficits, irregular borders and HGG-phenotype. Zone-2 patients showed a normal ARAT score at 2 months after surgery (Figure 5E). Permanent motor deficits (MRC = 4) were observed in 3 patients (4.5%), with previous treatment, HGG-phenotype, partial/subtotal resection (Table 2 and Table 3) and “distorted” cortical/subcortical maps.

When compared to the standard To5 technique, the advanced motor mapping technique, despite the increase in the rate of complete resection, did not increase the incidence of permanent motor deficits (Standard = 3.9% vs. Advanced = 4.5%, *p* = 0.869), either in the lower-grade setting (Standard = 3.3% vs. Advanced = 3.1%, *p* = 0.786) or in the high-grade one (Standard = 4.8% vs. Advanced = 5.6%, *p* = 0.651). Similarly, the incidence of permanent motor deficits was comparable in tumors with or without contrast enhancement, with both paradigms (Tumors with contrast enhancement, standard = 3.3% vs. Advanced = 4.5%, *p* = 0.973; Tumors without contrast enhancement, standard = 4.6% vs. Advanced = 4.5%, *p* = 0.795).

## 4. Discussion

Our study reports a novel technique for the on-line identification of high and low excitability M1 functional components during M1 tumors resection. This technique identifies the two functional components in all clinical conditions and disclosed different models of functional remodeling of M1 components, as well as their correlation with clinical and tumor-related parameters and patient motor performance. Preserving the high excitability component during resection significantly impacts on EOR and maintains a high rate of patient motor performance.

The intraoperative identification of two M1 functional components requires us to add to the qualitative approach currently practiced [11,12,13,15,16,17] the on-line measurement of many quantitative neurophysiological parameters: the number of pulses needed for effective stimulation, Motor-Threshold, MEP amplitude and latency. The evaluation of such parameters needs the combination of the “standard” HF-To5 paradigm and the reduced train (HF-To2) paradigm, in asleep anesthesia. We demonstrate that the on-line assessment of these parameters during surgery is feasible and provide the technical details to implement this technique in clinical practice. While not significantly increasing the mapping time, this novel technique requires the surgeon’s deep knowledge of motor system functional properties and his/her close coordination with a neurophysiology technician.

The technique identified the two functional components in all cases and clinical conditions. Three interesting elements emerged. In tumors outside M1 (involving or not CST), the functional rostro-caudal M1 organization was always preserved, with an overall depression of cortical excitability in large tumor masses. A “double picture” emerged, exploring tumors within M1: in 50% the functional cortical and subcortical architecture was preserved, while in the other half it was anatomically and somatotopically distorted, irrespectively of the tumors’ histo-molecular features or volume, and previous treatment was the parameter associated with this rearrangement. Whether “normal” or “distorted”, the two functional components were preserved and associated to presurgical normal motor abilities, suggesting a functional compensation whatever the M1 functional pattern developed [27]. The “distorted” map, not associated with pre-operative deficits, was correlated to poor seizure control, multiple AED uptake and previous treatment, suggesting that this rearrangement preserved motor performance, but with altered cortical excitability. Whether the latter resulted from the pattern of re-organization itself or whether it was the tumor inducing an imbalance of cortical excitability, in turn pushing towards a distorted reorganization, remains unsolved. Subcortical “distorted” maps always paralleled “cortical” distorted maps, except for two cases with a “distorted” subcortical map and a “normal” cortical map; should these be examples of incomplete reorganization, cortical rearrangement might be seen as the final stage of the action of the tumor on fibers altering the transmission to and from M1, finally changing its features [27].

Technically, the combination of HF-To5 and HF-To2 was mandatory for the identification of the two M1 components. However, at the cortical level, it was HF-To2 that identified the regions of lower excitability used to design the safe entry zone in which to perform the corticectomy. This area corresponded to the M1 anterior border region in most cases of “normal” map, while in “distorted”-map cases it was subject-dependent. Subcortically, HF-To5 and HF-To2 allowed for the identification of fibers with different excitability levels, evaluating the number of pulses needed for effective stimulation, current intensity (sMT), MEP amplitude and MEP latency. Again, it was HF-To2 that extended the resection, by allowing us to resect until an sMT of 3 mA, irrespectively of the lowest sMT obtained with HF-To5. In “normal” maps this resulted in a complete resection of the anterior (lowest excitability–slower) cortical and subcortical M1 region. The anterior M1 resection, was not associated to permanent deficits, but to motor-programming impairment and acute and fast recovering, suggesting a functional compensatory reshape [28,29]. The parallel distortion of somatotopic organization, in cases with a “distorted” map and a long clinical history, the fast recovery in most patients (74.5%) without rehabilitation (the latter limited to cases of short clinical history, pre-operative deficits, HGG phenotype) further supports this hypothesis [30]. Permanent deficits occurred only in 4.5% of cases, with previous deficits, a high grade or previous treatment, which constitutes a peculiar group of high-risk patients, possibly lacking the time of functional compensation, deserving a very careful resection.

This sophisticated mapping targeting the high excitability–fastest functional M1 component increased the resection, independently of clinical and imaging factors and cortical or subcortical map architectures: the high resolution of the technique guided the resections in any clinical, imaging or intra-operative functional condition, with tumor volume as the only limiting factor. The clinical relevance emerged when comparing results to those of patients operated with the standard technique, particularly in lower grades [12]. The significant increase in the rate of complete resection was associated with a high rate of preservation of patient functional capacity, which did not differ from that recorded in patients operated by the standard HF technique. This supports the conclusion that a better knowledge of the functional organization of M1 enhances the chance of achieving a larger tumor resection, possibly overcoming the limits of the traditional paradigm, which, being unable to disclose the components, often resulted in an early termination of the procedure.

This represents a major advancement in neuro-surgical oncology. However, it requires (1) a sophisticated IOM-machine capable of acquiring data in real-time and of shifting continuously between the different stimulation paradigms; (2) a dedicated well-trained neurophysiologist/technician for rapid response interpretation, (3) a surgeon highly-trained in intraoperative neurophysiology, able to interpret responses and consequently adopt appropriate surgical actions; (4) a specific learning curve. Resections were always performed under general anesthesia, providing the stable cortical excitability (more variable in awake conditions) needed for the on-line interpretation of the quantitative parameters—specifically MEP amplitude, cMT and sMT, MEP latency [10,11]—needed for the on-line identification of the two M1 functional components. The level of anesthesia, monitored by ECoG, should not be too deep, to avoid masking the responses [10,11,31]. Furthermore, the continuous assessment of CST integrity by continuous MEP monitoring is also recommended [10,11,31]. These technical requirements are mandatory to adopt the technique in clinical practice.

The surgical results also add elements of discussion on the possible distinct functional roles of the two M1 components. Upon identification of the two M1 functional components at the cortical and subcortical levels, higher excitability fibers are always preserved and lower excitability sites removed, leading to a favorable motor outcome. Data in humans are very limited [9,32]. Recent studies in awake patients demonstrated that the stimulation of the two components during the performance of a hand-manipulation task induces different motor interferences: muscle activation (posterior) vs. complete suppression or dysfunctional recruitment of muscles (anterior) [9]. Diffusion tractography revealed U-shaped fibers connecting the anterior sector to premotor areas and the posterior sector to S1 [9,33] and suggests a role of the anterior sector in mediating motor planning and execution, as a likely caudal extension of the dorsal premotor areas and a role of the posterior sector in on-going tactile and somato-sensory feedback in the executive stage of movement [9,33,34]. The preservation of a higher excitability and fastest fibers (posterior)—those in monkeys monosynaptically connected with motoneurons—is crucial for movement performance and dexterity. These are preserved while the resection of lower excitability sites, indirectly connected with motoneurons, results in a transient deficit of action performance, supporting their involvement in the sensori-motor integration required by the motor program. The fast recovery, however, suggests the existence of an accessory system compensating for the loss of the anterior component. Dedicated studies should address this issue.

## 5. Conclusions

In conclusion, the novel mapping technique advanced allows us to distinguish, in real time, the two M1 functional components and preserve the highest excitability one in the clinical routine. On the one hand, this affords a high rate of EOR and maintains a global safe motor outcome, independent of the clinical context; on the other hand, it suggests an intrinsic M1 plasticity potential.

## Figures and Tables

**Table 1:** Clinical, imaging, intraoperative and post-operative findings in the 66 patients with a tumor within M1.

*N*. 66	Variables	Value	%	Variables		Value	%
Clinical and Demographic Features							
Sex	Female	28	42.4	Age, years	Mean	45.5	
	Male	38	57.6		Median	44.2	
Focal seizure at onset	Yes	60	90.9	Duration of clinical history	>6 mo	49	74.2
	No *	6	9.1		<6 mo	17	25.8
Previous treatment	No	33	50	Pre-operative motor deficit	No	55	83.3
	Yes	33	50		Yes	11	16.7
Controlled pre-op seizures	No	41	62.1	Side	Right	35	53%
	Yes	25	37.9		Left	31	47%
Radiological Features							
Tumor volume, cm^3^	Mean	6.66		Residual volume, cm^3^	Mean	0.45	
	Median	3.74			SD	1.93	
Resection	Partial	8	12.0	EOR	Mean	97.1	
	Total	58	88.0		SD	9.5	
Cortical outcrop	No	18	27.3	Contrast-enhancing lesion	No	44	66.7
	Yes	48	72.7		Yes	22	33.3
Border	Irregular	50	75.8	Tumor extension outside M1	No	52	78.8
	Defined	16	25.2		Yes	14	21.2
Site	M1	53	80.3	Berger classification **	1	31	47
	M1 dPM	6	9.1		2	25	37.9
	M1-S1	4	6.1		3	10	15.2
	M1-SMA	3	4.5				
Intraoperative Findings							
Cortical pattern	Normal	36	54.5	Subcortical pattern	Normal	34	51,50%
	Distorted	30	45.5		Distorted	32 #	48.5
Histo-Molecular Profile							
Histology	LGG	36	54.5	IDH 1-2	Mutated	32	48.5
	HGG	30	45.5		Wildtype	34	51.5
Codeletion 1p/19q	No	50	75.8				
	Yes	16	24.2				
Outcome							
5-days motor deficit	No	15	22.7	1-mo motor deficit	No	63	95.5
	Yes	51	77.3		Yes	3	4.5

* The remaining 6 cases had generalized seizures. ** classification of zones of the motor cortex based on Berger: zone 1 = lower limb, zone 2 = upper limb, zone 3 = face; # The 2 patients with a normal cortical map and a distorted subcortical map had a tumor deep-seated in M1; Mo = months; M1 = primary motor area; dPM = dorsal Pre-Motor area; S1 = primary sensory area; SMA = supplementary motor area; LGG = Lower grade glioma; HGG = Higher grade glioma IDH = Isocitrate dehydrogenase; SD = Standard Deviation.

**Table 2:** Variables associated with the cortical or subcortical pattern, motor deficit and extent of resection: Fisher Exact tests or Pearson Chi-square tests were performed for all the associations, with the exception of the tumor volume variable, where a one-way ANOVA was used; only significant factors are shown. The Advanced Brain Mapping Technique analysis refers to the comparison of EOR between patients submitted to the advanced Brain Mapping technique (YES) and those (historical cohort of 51 patients) who were submitted to the standard mapping (NO, To5).

Factors Associated with Cortical Pattern								Factors Associated with Subcortical Pattern							
		Normal	%	Distorted	%		*p*				Normal	%	Distorted	%	*p*
Previous	No	23	69.7	10	30.3		0.01	Previous		No	22	66.7	11	33.3	0.01
treatment	Yes	13	39.4	20	60.6			treatment		Yes	12	36.4	21	63.6	
Controlled	Yes	27	65.9	14	34.1		0.02	Controlled pre-op seizures		Yes	25	61	16	39	0.04
pre-op seizures	No	9	36	16	64					No	9	36	16	64	
Subcortical pattern	normal	34	100	0	0		0.001								
	distorted	2	7	30	93										
FACTORS ASSOCIATED WITH MOTOR DEFICIT AT 5 DAYS								FACTORS ASSOCIATED WITH MOTOR DEFICIT AT 1 MONTH							
		DEFICIT									DEFICIT				
		Yes	%	No	%		*p*				Yes	%	No	%	*p*
Berger Classification	1	19	61	12	39		0.01	Resection		Partial/Subtotal	2	25	6	75	0.03
	2	23	92	2	8					Total	1	2	57	98	
	3	9	90	1	10										
Border	Irregular	44	88	6	12		0.001								
	Well-defined	7	44	9	56										
Histology	LGG	34	94	2	6		0.001								
	HGG	13	43	17	57										
FACTORS ASSOCIATED WITH MRC AT 5 DAYS								FACTORS ASSOCIATED WITH MRC AT 1 MONTH							
		MRC	%	MRC	%	MRC	%	*p*			MRC	%	MRC	%	*p*
		3		4		5					4		5		
Duration	>6m	13	26.5	32	6.3	4	8.2	0.04	Resection	Partial/ Subtotal	3	38	5	62	0
	<6m	9	52.9	5	29.4	3	17.6			Total	1	2	57	98	
Border	Irregular	21	42	27	54	2	4	0.01	Histology	LGG	0	0	36	100	0.04
	Defined	1	6.3	10	62.5	5	31.3			HGG	4	13	26	87	
FACTORS ASSOCIATED WITH EOR															
		Partial/Subtotal	d.s/%	Total	d.s/%		*p*	Cortical pattern		normal	0	0	36	100	0.04
Volume pre-op	mean	24.5	5.6	4.7	4.3		0.01			distorted	4	13	26	87	
Advanced Brain Mapping Technique	No	14	27	37	73		0.03	Subcortical pattern		normal	0	0	34	100	0.04
	Yes	8	12	58	88					distorted	4	13	28	87.1	

**Table 3:** Logistic regression. Significant predictors of Cortical Pattern, Subcortical Pattern, Motor performances at 5 days and 1 month, and Extent of resection. Histo-molecular profile (HGG); Duration of Clinical History (>6 m); Borders (Well-demarcated); EOR classes (Total vs. Partial/Subtotal).

Predictors/	Cortical Pattern			
Dependent Variables				
	Coefficients	S.E.	*p* value	Exp(B)
Previous Treatment	1.264	0.520	0.015	3.538
Seizure control	1.232	0.531	0.020	3.429
Subcortical Pattern	6.171	1.251	0.001	478.0
	Subcortical Pattern			
	Coefficients	S.E.	*p* value	Exp(B)
Previous Treatment	1.253	0.517	0.015	4.025
Cortical Pattern	6.171	1.251	0.001	478
	Pre-operative Deficits			
	Coefficients	S.E.	*p* value	Exp(B)
Cortical Pattern	−1.386	1.275	0.06	4.000
Histo-molecular profile	−1.386	1.275	0.06	4.000
	Deficits at 5 days			
	Coefficients	S.E.	*p* value	Exp(B)
Borders	2.244	0.666	0.001	9.429
Histo-molecular profile	−2.565	0.816	0.002	0.077
	Deficits at 1 month			
	Coefficients	S.E.	*p* value	Exp(B)
EOR classes	−2.944	1.298	0.023	0.053
	MRC at 5 days			
	Coefficients	S.E.	*p* value	Exp(B)
Duration of Clinical History	−1.136	0.584	0.048	0.321
Borders	−2.385	1.072	0.026	0.092
	MRC at 1 month			
EOR Classes	3.532	1.245	0.005	34.2
	Extent of Resection			
Variables	Coefficients	S.E.	*p* value	Exp(B)
Volume	−1.009	0.491	0.040	0.365

## Data Availability

Data can be requested upon reasonable request.

## Supplementary Materials

The following are available online at https://www.mdpi.com/article/10.3390/cancers13153808/s1, Text S1, Figure S1: Example of motor responses obtained by HF-To5 and HF-To2, Table S1: main clinical and imaging features of patients belonging to the group: M1 intact and CST not involved, or M1 intact and CST involved (at a variable extent); Table S2: Clinical, imaging, intraoperative and post-operative findings of the 51 patients with a tumor within M1 operated with the aid of the standard mapping technique (To5). Video S1: Cortical and subcortical mapping performed with the aid of the advanced motor mapping technique in a case of low-grade glioma (right hemisphere) with normal cortical and subcortical maps; Video S2: Subcortical mapping performed with the aid of the advanced motor mapping technique in a case of low-grade glioma (left hemisphere) with distorted cortical and subcortical maps; Video S3: Finger movements recorded during the first post-operative day in a patient with an M1 tumor and distorted cortical and subcortical maps; Video S4: Example of a task (ARAT) recorded during the second post-operative day in a patient with an M1 tumor and distorted cortical and subcortical maps.
