# Supplementary material for: Targeting Primary Motor Cortex (M1) Functional Components in M1 Gliomas Enhances Safe Resection and Reveals M1 Plasticity Potentials
Source: Cancers (Basel). 2021 Jul 28;13(15):3808. doi: 10.3390/cancers13153808 (PMC8345096; doi:10.3390/cancers13153808)
Supplement: Supplementary file 1 [file cancers-13-03808-s001.zip › cancers-1269321-supplementary.pdf]

Supplementary Materials: Targeting Primary Motor Cortex (M1) Functional Components in M1 Gliomas Enhances Safe Resection and Reveals M1 Plasticity Potentials

Marco Rossi, Luca Viganò, Gugliemo Puglisi, Marco Conti Nibali, Antonella Leonetti, Lorenzo Gay, Tommaso Sciortino, Luca Fornia, Vincenzo Callipo, Marta Lamperti, Marco Riva, Gabriella Cerri and Lorenzo Bello

Text S1: Suppl. Methods

The following muscles were recorded: EDC (Extensor-Digitorum), FDI (First-Dorsal-Interosseum), APB (Abductor-Pollicis-Brevis), ADM (Abductor-Digiti-Minimi), FCR (Flexor-Carpis-Radialis), Quadriceps, TA (Tibialis-Anterior), FHC (Flexor-Hallucis-Brevis), Orbicularis Oris (bilateral), Tongue (bilateral), Mentalis (Bilateral), Cricothyroid (Bilateral), Biceps Brachiis (Bilateral).

ARAT is composed by 19 items subdivided in 4 subscales, with 3 to 6 tasks each. Each task runs until the subject completes the task or until reaching a time limit of 60 seconds. The quality of the task is rated on an ordinal 4 point-scale, that is, 0 (movement impossible), 1 (partially completes the task within the 60 second), 2 (completed but either with great difficulty or takes more than 5 seconds) , or 3 (normal movement performed in less than 5 seconds). The maximum score for the ARAT is 57 with a higher score indicating better arm motor performance. In grasp tasks the subject is asked to to grasp, lift vertically, place, and then release each three objects (block, ball, or stone). In the grip tasks subject is asked to pour water from one cup to the other or to horizontally displace 2 different sized alloy tubes. In the pinch tasks subject is asked to grasp a ball bearing or a marble from a tin lid, lift it up vertically, then place and release it into a target tin lid placed on the shelf. In the gross movement tasks the subject is asked to move the shoulder and elbow across a wide range of motion with accompanying forearm movement (See [1]).

Tumor volume was calculated using Smartbrush-software-Elements-BrainLab; for contrast-enhancing lesions, the contrast-enhancing portion of the tumor (target of resection) was measured [10,12]. For lower-grade/non–contrast-enhancing tumors, FLAIR signal (target of resection) was used. For zone location, M1 was divided in three zones (zone-1: lower limb; zone-2: upper limb; zone-3: face) [10,12]. Tumor borders were defined as well-defined or irregular [10,25], using post-contrast imaging for contrast lesions, or FLAIR for non-enhancing. Outcrop of M1 was defined as present when the tumor was reaching the surface, either on coronal and/or sagittal FLAIR for non-enhancing lesions, or on T1-post-contrast images for enhancing lesions [10,12]. Regarding the extension of the tumor, only tumors with over 75% of their mass involving M1 were included; in cases of large tumor volume, the tumor could extend anteriorly toward the dorsal-premotor or the supplementary-motor cortex or posteriorly toward the primary-sensory cortex. The extension was categorized accordingly [10,12].

EOR corresponded to the percentage of volume resected with respect to the preoperative volume: (preoperative volume − postoperative volume)/preoperative volume. Surgical resection was categorized as: i) total resection (postoperative volume = 0 cm^3), ii) subtotal resection (postoperative volume 0 < 5 cm^3 and iii) partial resection (postoperative volume > 5 cm^3 [10,12,21].

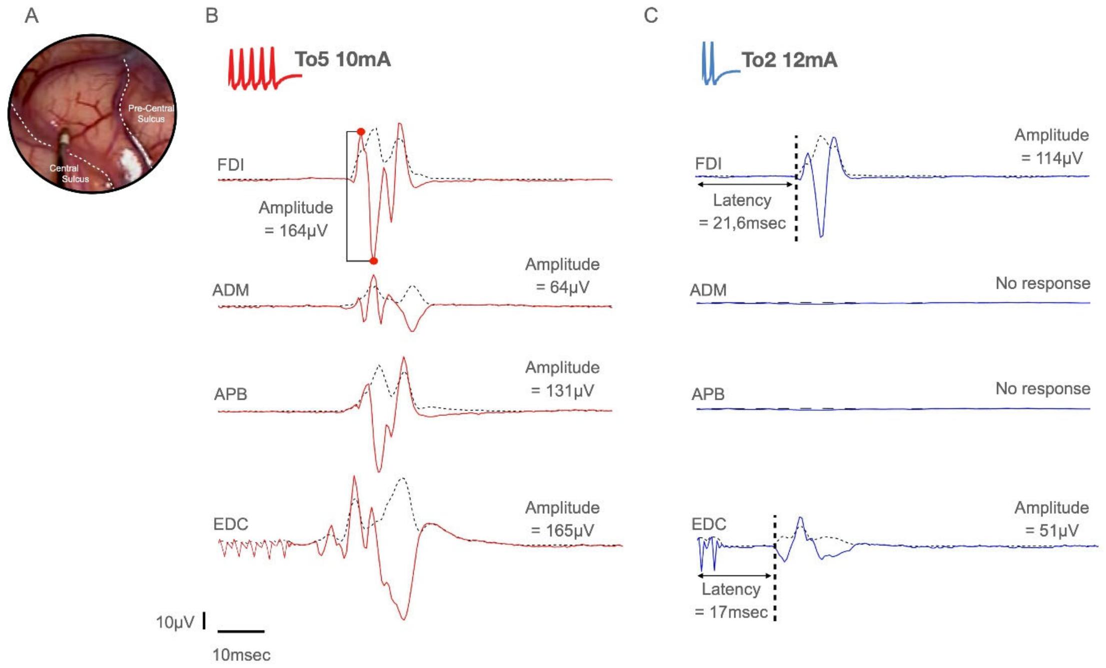

Figure S1. Example of motor responses obtained by HF-To5 and HF-To2. The same site (**A**) located at the posterior bank of the right hemisphere hand-knob was stimulated initially by HF-To5 followed by HF-To2, delivered by a monopolar probe, in an anesthetized patient. In (**B**) the MEPs evoked by HF-To5 at cMT (10 mA) are reported. To5 evoked responses from FDI (flexor allucis brevis), ADM (abductor digiti minimi), APB (abductor pollicis brevis) and EDC (Extensor digiti); the amplitude (indicated by a continuous black line) of each MEP is reported. All MEPs were polyphasic. In (**C**) the MEPs evoked by HF-To2 at cMT (12 mA) are shown. HF-To2 evoked responses from FDI and EDC only. The amplitude of each MEP is reported, along with the MEP latency (calculated from the stimulus onset, indicated with an arrow and the MEP onset, indicated by a perpendicular dotted line). FDI MEP is monophasic, EDC polyphasic. In the EDC trace the stimulus artifact (at the beginning of the trace) is also visible.

Table S1. main clinical and imaging features of patients belonging to the group: M1 intact and CST not involved, or M1 intact and CST involved (at variable extent).

**M1 Intact, CST Not Involved**	***N***	**Location**	**Previous Deficits**	**Volume**
9	4 LGG<br>5 HGG	Left Temporal 3<br>Right Temporal 6	NO	Mean 7.33 cc<br>Median 5.33 cc
Age. 48 yrs (34–67)				
Sex. Male 4<br>Female 5				
**M1 Intact, CST Involved**	***N***	**Location**	**Previous Deficits**	**Volume**
28	19 LGG<br>9 HGG	Anterior 19<br>10 Left<br>9 Right<br>(premotor, SMA)	YES 2<br>1<br>1	Mean 11.45 cc<br>Median 14.33 cc
Age. 42 yrs (31–59)		Posterior 2<br>(parietal)	-	5 large tumors<br>(2 anterior, 3 insula)
Sex. Male 13<br>Female 15		Deep (insula) 7	-	

LGG = Low Grade Glioma; HGG = High Grade Glioma; Premotor = Premotor Area; SMA = Supplementary Motor Area

Table S2. Clinical, Imaging, intraoperative, and post-operative findings of the 51 patients with a tumor within M1 operated with the aid of the standard mapping technique (To5).

***N.* 51**	**Heading Title**	**Value**	**%**	**Heading Title**	**Heading Title**	**Value**	**%**
Clinical and demographic features							
Sex	Female	24	47.0%	Age, yrs	Mean	42.5	
	Male	27	53.0%		Median	41.2	
Focal seizure at onset	Yes	45	88.2%	Duration of clinical history	>6 mo	32	62.7%
	No*	6	11.8%		<6 mo	19	37.3%
Previous treatment	No	36	70.5%	Pre-operative motor deficit	No	46	90.1%
	Yes	15	29.5%		Yes	5	9.9%
Controlled pre-op seizures	No	32	62.7%	Side	Right	29	56.8%
	Yes	19	37.3%		Left	22	43.2%
Radiological features							
Tumor volume, cm3	Mean	6.89		Residual volume, cm3	Mean	3.95	
	Median	4.01			DS	2.98	
Resection	Partial Subtotal	14	27%	EOR	Mean	71.1%	
	Total	37	73%		DS	4.5	
Cortical outcrop	No	8	15.7%	Contrast-enhancing lesion	No	30	58.8%
	Yes	43	84.3%		Yes	21	41.2%
Border	Irregular	34	66.7%	Tumor extension outside M1	No	39	76.4%
	Defined	17	33.3%		Yes	12	23.6%
Site	M1	39	76.4%	Berger classification**	1	7	13.7%
	M1 dPM	3	5.8%		2	31	60.7%
	M1–S1	7	13.7%		3	13	25.4%
	M1–SMA	2	3.9%				
Histo-molecular profile							
Histology	LGG	22	43.1%	IDH 1–2	Mutated	30	58.8%
	HGG	29	56.9%		Wildtype	21	41.2%
Codeletion 1p/19q	No	42	82.3%				
	Yes	9	17.6%				
Outcome							
5-days motor deficit	No	2	3.9%	1-mo motor deficit	No	49	96.1%
	Yes	49	96.1%		Yes	2	3.9%

* The remaining 6 cases had generalized seizures. ** classification of zones of the motor cortex based on Berger: zone 1 = lower limb, zone 2 = upper limb, zone 3= face; mo = MonthsS; Mo = months; M1 = primary motor area; dPM = dorsal Pre-Motor area; S1 = primary sensory area; SMA = supplementary motor area; LGG = Lower grade glioma; HGG = Higher grade glioma IDH = Isocitrate dehydrogenase.

VIDEO 1: Cortical and subcortical mapping performed with the aid of the advanced motor mapping technique in a case of low-grade glioma (right hemisphere) with normal cortical and subcortical map.

VIDEO 2: Subcortical mapping performed with the aid of the advanced motor mapping technique in a case of low-grade glioma (left hemisphere) with distorted cortical and subcortical map.

VIDEO 3: Finger movements recorded during the first post-operative day in a patient with a M1 tumor and distorted cortical and subcortical map.

VIDEO 4: Example of task (ARAT) recorded during the second post-operative day in a patient with a M1 tumor and distorted cortical and subcortical map. The patient is performing one of the grip sub-tasks: the task at hand requires displacing an alloy tube from a starting position to a target peg on a plank. Assigned score: 2 out 3 (completed in more than 5 seconds). The total score of the patients was 56 in the preoperative phase and 54 in the postoperative evaluation**.**

References:

1. Yozbatiran, N.; Der-Yeghiaian, L.; Cramer, S.C. A Standardized Approach to Performing the Action Research Arm Test. *Neurorehabilit. Neural Repair* **2007**, *22*, 78–90, doi:10.1177/1545968307305353.
